# Exploring the Risk: Peripheral Retinal Degenerations in Young Australian Adults

**DOI:** 10.3390/jcm14103501

**Published:** 2025-05-16

**Authors:** Natalie Ann Watt, Nicholas Hockley, James Andrew Armitage

**Affiliations:** School of Medicine (Optometry), Faculty of Health, Deakin University, 75 Pigdons Road, Geelong 3216, Australia

**Keywords:** peripheral retinal degeneration, refractive error, axial length, retinal detachment, ultra-widefield retinal imaging, Zeiss Clarus™ 500

## Abstract

**Background/Objectives:** Peripheral retinal degenerations (PRDs) are structural anomalies in the outer regions of the retina, typically emerging in adolescence and early adulthood. Early detection is crucial, as some PRDs can lead to sight-threatening complications, such as retinal detachment, if left unmanaged. Due to a paucity of research regarding PRDs and their association with axial length (AL) and refractive error (RE) in young Australian adults, this study aimed to investigate the prevalence of PRDs in this population and establish whether AL and RE could help predict the likelihood of PRD occurrence. **Methods**: A cross-sectional study was conducted on a mixed population (*n* = 221) of Australian adults aged 18 to 40. Demographic data, RE, AL, and a series of ultra-widefield (UWF) retinal images were obtained from participants’ undilated eyes using the Zeiss Clarus^TM^ 500. **Results**: The overall PRD prevalence was 8.15% (*n* = 442 eyes). Binary logistic regression revealed that a longer AL was a more significant factor in increasing the risk of PRD development across all myopia classifications compared to emmetropia than RE. The likelihood of a PRD was 50% at an AL of 26.9 mm and −6.50D of myopia, and 95% at 29.6 mm and −11.00D. **Conclusions**: PRD prevalence was lower than reported in other global studies, perhaps reflecting the diverse ethnic makeup of the cohort. While our study supports the conventional understanding that longer ALs, and high myopia are key risk factors for developing a PRD, it also provides new insights into the likelihood of detecting a PRD at a given AL or RE in a mixed population. This information is crucial for eye care practitioners, enabling early identification of at-risk individuals and screening for PRDs that may increase the risk of retinal detachment.

## 1. Introduction

Peripheral retinal degenerations (PRDs) comprise a broad group of abnormalities affecting the peripheral retina and choroid. While some PRDs, such as White Without Pressure (WWOP), and Dark Without Pressure (DWOP), are often considered clinically benign, others, most notably lattice degeneration (LD), retinal holes and tears, can increase the risk of retinal detachment. Reported prevalence rates of PRDs vary widely across studies, ranging from approximately 7% to 62% [1,2]. These degenerations typically develop during the second and third decades of life, with the risk of a retinal detachment increasing significantly with age, particularly in cases involving LD [3,4]. Therefore, early detection and ongoing monitoring of PRDs in younger adults is essential for reducing the risk of vision loss associated with retinal detachment.

To date, most population-based studies investigating PRDs have been conducted on Asian populations with myopia, with findings indicating a strong association between PRDs, high myopia, and longer ALs [2,5,6,7,8].

At birth, the average axial length (AL) is approximately 16 mm, increasing to about 24 mm by early adulthood [9], with further elongation associated with the progression of myopia and a range of retinal pathologies. Research findings indicate that Southeast Asian subjects with ALs > 26 mm have a significantly higher odds ratio (OR) of developing a PRD (OR:3.37—any peripheral change, OR:2.93-WWOP and OR:1.44-LD) [2,6], while ALs ≥ 30 mm are positively correlated with retinal holes [10]. Given that longer ALs are considered a strong predictor for PRD development, measuring AL could serve as a key biometric parameter for eye care practitioners to assess individuals at higher risk for PRDs.

Although PRDs are less frequently observed in hyperopic and emmetropic eyes, WWOP has been reported in up to 7% of hyperopes and 5% of emmetropes among young Southeast Asian adults [8]. Degenerative retinoschisis, which is linked with hyperopia, affects approximately 5% of the population over the age of 20 [11,12], while LD has been reported in 1.4% of emmetropes [8]. While several studies have explored the relationship between AL, refractive error (RE) and PRDs in Southeast Asian cohorts, to the best of our knowledge, research on this topic in young Australian adults with diverse ethnic backgrounds remains limited. Given the potential risk of sight-threatening complications associated with certain PRDs, further research into the relationship between PRDs, AL, and RE could greatly assist eye care practitioners in identifying high-risk individuals at an earlier age and implement preventative management strategies.

The gold standard for evaluating the mid and far peripheral retina, defined as the field of view between 60 and 220° [13], is a dilated fundus examination (DFE) with scleral indentation [14]. However, in some sectors, Australian optometrists do not routinely dilate their patients without a clinical indication. A refractive error greater than −4.00D is used by many as this clinical indication. This may be due to factors such as the additional consultation time required or the temporary visual impairment it causes, which can be inconvenient for patients. With the advent of ultra-widefield (UWF) imaging techniques, it is now possible to capture more than 80% (200°) of the retina swiftly without mydriasis [15]. This field of view is comparable to what an experienced eye care practitioner can achieve with a DFE and makes UWF imaging a very useful adjunct for screening and detecting potentially sight-threatening retinal conditions, most of which are found within the mid-peripheral 60–200° area.

As UWF imaging becomes increasingly integrated into clinical practice in Australia, it could serve as a valuable screening tool in assessing the peripheral retina, particularly in cases where a DFE is not performed. This would then be the impetus for the ‘gold standard’ undilated examination.

This study aims to determine the prevalence of peripheral retinal degenerations in a young Australian adult population and to assess the relationship between PRD, AL, and RE, as well as their potential to predict the likelihood of a PRD being present.

## 2. Materials and Methods

### 2.1. Participant Recruitment

Two hundred and twenty-one participants (442 eyes) from a mixed-ethnicity population (Caucasian, Mediterranean, Sub-Saharan African, Indian subcontinental, Southeast Asian) were recruited via flyers posted on Deakin University Waurn Ponds optometry noticeboards between April 2022 and August 2024. Eligibility criteria required participants to be at least 18 years old, for consent purposes, and no older than 40 years, as this age range was used to define a ‘young adult’ [16].

Those participants undergoing orthokeratology treatment or who had previously undergone refractive surgery were excluded, as both treatments affect AL measurements and the relationship between AL and RE. Although low-dose atropine treatment for myopia control may also influence AL, it was not included in the exclusion criteria. Additionally, participants with media opacities likely to compromise the quality of ultra-widefield retinal imaging were excluded.

### 2.2. Study Design

This study employed a cross-sectional design, selected for its ability to examine a representative cross-section of the population and generate findings that could be generalised to the entire target population. Each participant attended a single session, during which demographic data (including age and gender information) were collected. RE and AL measurements were obtained for both eyes using the Lenstar^®^ LS 900 (Haag-Streit, Köniz, Switzerland) and an open-field autorefractor (NVision-K 5001, Shin-Nippon, Tokyo, Japan), respectively. The average of three AL readings was recorded in millimetres (mm), while RE measurements were recorded in dioptres (D). A four-image auto-montage (superior, inferior, nasal, and temporal) UWF digital image was captured for both eyes (without dilation), using the Clarus 500™ (Carl Zeiss Meditec Inc., Dublin, CA, USA).

### 2.3. Data Preparation

UWF images captured with the Clarus^TM^ 500 were downloaded, and two principal researchers independently assessed the images to identify any peripheral retinal degenerations. In cases of disagreement, consensus was reached through discussion, or the third principal investigator was consulted.

All peripheral retinal changes were classified and documented in an Excel spreadsheet using Microsoft^®^ Excel^®^ for Microsoft 365 (Version 2409 Build 16.0.18025.20160).

Retinal changes not commonly associated with retinal detachment, including Congenital Hypertrophy of the Retinal Pigment Epithelium (CHRPE) and Choroidal Naevi [17,18,19], were also classified, but not included in the overall PRD statistical analysis. While WWOP and DWOP typically have minimal retinal complications, their potential retinal detachment risk warrants consideration. WWOP, in particular, can be associated with LD [20], and both WWOP and DWOP are linked to longer ALs and high myopia—two key risk factors for retinal detachment [7,20].

Both eyes were included in the analysis to obtain the most accurate estimate of PRD prevalence, as PRDs can occur unilaterally or bilaterally [11,21,22]. Each identified PRD was assigned a value of 1, while the absence of a PRD was recorded as 0. These data, along with demographic information (age and gender), AL, and RE measurements, were also recorded in the Excel spreadsheet. RE measurements were converted to spherical equivalent in dioptres (D) by adding the sum of the sphere power with half of the negative cylinder power and classified based on criteria from previous studies (Table 1) [23,24,25,26,27,28]. AL values were also binned and classified as per Khan et al. [29] (Table 2).

The Excel spreadsheet data were transferred to IBM SPSS Statistics (Version 29.0.2.0, IBM Corp., Armonk, NY, USA) for further statistical analysis.

### 2.4. Statistical Analysis

Descriptive statistics, Chi-square (χ^2^) and independent *t*-tests were conducted in SPSS to analyse the data. Binary logistic regression was used to evaluate the likelihood and OR for the presence of a PRD and the variables AL and RE. Logistic regression coefficients and intercept values obtained from SPSS were further utilised in Excel to calculate curve values, facilitating the plotting of logistic regression graphs. A significance level of *p* = 0.05 was applied to all tests.

### 2.5. Ethical Considerations

This study adhered to the tenets of the Declaration of Helsinki. Ethics approval was granted by Deakin University’s Faculty of Health Human Ethics Advisory Group (HEAG_H 15_2022) on 29 March 2022. Participants were all given a hard copy of the plain language statement and provided written consent for research participation before data were collected. Upon enrolment, participants were allocated a unique identifier to ensure all data remained anonymous. Referrals to an external eye care practitioner were provided for all cases where a PRD was noted, in order for participants to seek further independent clinical advice.

## 3. Results

### 3.1. Demographic Comparisons

There were 150 male eyes (33.9%) and 292 female eyes (66.1%) analysed in the study. The mean age of participants was 22.43 ± 3.11 years (range 18–39 years old), with males averaging 22.52 ± 3.17 years and females 22.38 ± 3.10 years.

Participants without a PRD had a mean age of 22.43 ± 3.15 years, while for those with a PRD, it was 22.39 ± 2.74 years (Table 3). Overall, no significant association was found between gender (χ^2^ (1) = 2.399, *p* = 0.121) or age (t(440) = 0.073, *p* < 0.942) and the presence of a PRD. The mean RE did not differ significantly between males and females (t(440) = −1.53, *p* < 0.063); however, males had a slightly longer mean AL (24.45 ± 1.17 mm) compared to females (23.80 ± 1.11 mm), (t(440) = 5.714, *p* < 0.001).

### 3.2. Peripheral Retinal Degeneration Prevalence

**Figure 1:**
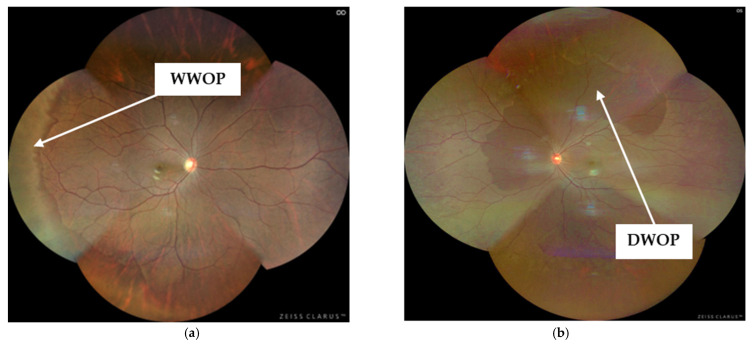
(**a**). Clarus™500 image (WWOP) (**b**). Clarus™500 image (DWOP).

PRDs were detected in thirty-six eyes (8.15%), with eight eyes from males (1.81%) and twenty-eight from females (6.33%). Among the 442 eyes analysed, the PRDs identified were WWOP, DWOP, LD and a retinal hole. The most frequently observed PRDs were WWOP (Figure 1a) and DWOP (Figure 1b), each occurring in 3.39% (*n* = 16/442) of cases (Table 4). One participant had two different PRDs identified in the same eye, accounting for the thirty-seven PRDs reported in Table 4.

Table 5 displays the PRD frequencies associated with AL and RE. PRD prevalence was 0.61% (*n* = 1/165) in emmetropes, 2.86% (*n* = 1/35) in hyperopes, and 14.05% (*n* = 34/242) in myopes. Within the myopic group, PRD prevalence was 3.95% (*n* = 6/152) for mild myopia, 22.06% (*n* = 15/68) for moderate myopia, and 59.09% (*n* = 13/22) for high myopia. For AL, PRD prevalence was 15.23% (*n* = 30/197) for eyes longer than the mean AL of 24.02 ± 1.17 mm.

### 3.3. Association Between Refractive Error and Peripheral Retinal Degenerations

The overall mean RE was −1.43 ± 2.21D. For eyes without a PRD, the mean RE was −1.13 ± 1.94D, while for eyes with a PRD, it was −4.78 ± 2.33D. The largest hyperopic RE observed with a PRD was +0.75D, while the lowest for myopia was −1.00D. For the emmetropic eyes, one PRD was noted at +0.50D. PRDs were significantly associated with a moderate to high myopic RE (t(10.62) = 0.035, *p* < 0.001) (Table 6).

### 3.4. Association Between Axial Length and Peripheral Retinal Degenerations

For eyes without a PRD, the mean AL was 23.89 ± 1.08 mm, whereas for eyes with a PRD, it was 25.46 ± 1.13 mm. Whilst the shortest AL with a PRD was 22.96 mm, PRDs were significantly associated with longer axial lengths (t(440) = 0.643, *p* < 0.001) (Table 6).

### 3.5. Binary Logistic Regression Analysis Between Axial Length, Spherical Equivalent Refractive Error and Peripheral Retinal Degenerations

Binary logistic regression analysis showed that the risk of having a PRD compared to emmetropia significantly increased with higher degrees of myopia (moderate myopia OR:3.84; 95% CI, 1.79–5.86; *p* < 0.001, high myopia OR:5.47; 95% CI, 3.33–7.61; *p* < 0.001) (Table 7). Moreover, PRD risk also increased with axial elongation > 24.50 mm and remained statistically significant across all AL classifications-mild, moderate and high myopia (*p* < 0.001) compared with ALs between 23.50 mm and 24.50 mm (emmetropia). Interestingly, the OR for ALs over 26.50 mm (high myopia) was slightly lower than that for ALs between 25.50 mm and 26.50 mm (moderate myopia) (Table 7).

**Figure 2:**
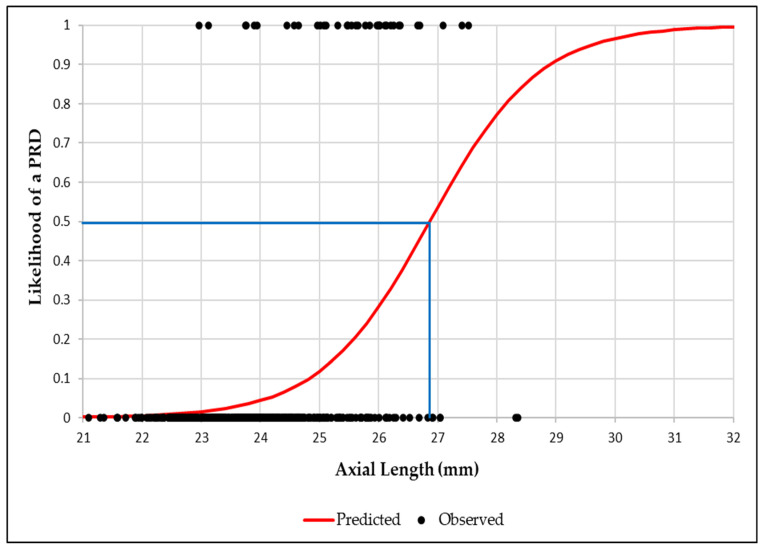
Likelihood of peripheral retinal degeneration (PRD) versus axial length (50% likelihood illustrated by the blue line).

**Figure 3:**
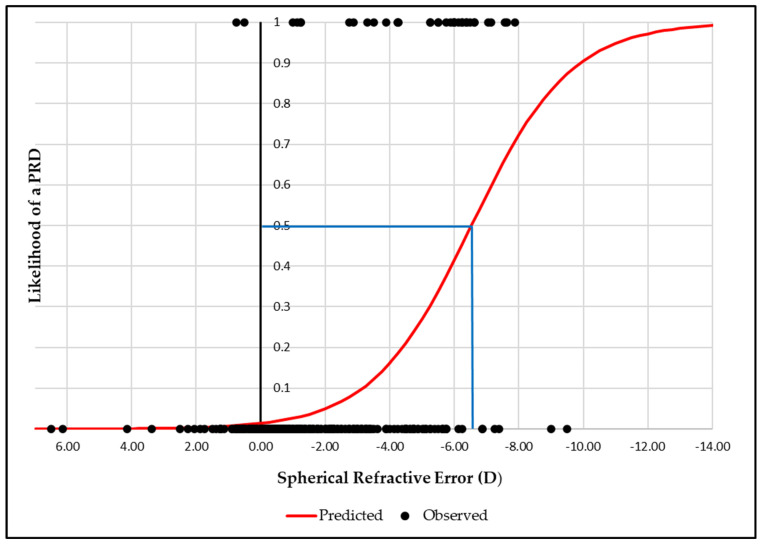
Likelihood of peripheral retinal degeneration (PRD) versus spherical refractive error (50% likelihood illustrated by the blue line).

Binary logistic regression graphs showed that the likelihood of a PRD reached 50% at an AL of 26.9 mm, increasing to 95% at 29.6 mm (Figure 2). Similarly, for RE, a 50% likelihood was observed at −6.50D, rising to 95% at −11.00D (Figure 3).

## 4. Discussion

The clinical significance of PRDs stems from their association with having an increased risk of retinal detachment. Although many PRDs, such as WWOP and DWOP, are not directly linked to retinal detachment, unlike LD or retinal holes, their potential risk still merits attention. This is because WWOP is associated with LD [17], and both WWOP and DWOP are connected to longer ALs and high myopia—two major risk factors for retinal detachment development [7,17].

In this study, PRDs were identified in 8.15% of eyes among young Australian adults aged 18 to 40 years from a diverse ethnic background. Although the literature regarding PRDs in this age group remains somewhat limited, our findings closely align with Michaud and Forcier [1], who reported a prevalence of 7.74% in the Canadian adult population, a country with similar demographics to Australia [30]. Higher prevalence rates have been reported in other studies; however, direct comparisons to this study are challenging due to differences in study populations. Many of these studies included older participants, a Southeast Asian demographic and/or exclusively recruited myopic individuals—a group known to have an increased predisposition to developing PRDs [2,6,7,8].

The most frequently observed PRDs in this study were WWOP and DWOP, each with a prevalence of 3.39%. This is lower than the prevalence rates reported in the current literature, which range from approximately 6% to 46% [6,7,8,21,31] for WWOP and 30% for DWOP [7]. However, as previously mentioned, many of these studies predominantly recruited participants with high myopia, which may limit the generalisability of their findings to the broader population. Dhull et al. [21], reported a WWOP prevalence rate of 6.11%, a value closer to the findings of our study. This similarity is likely attributable to the inclusion of a more balanced distribution of refractive errors incorporating myopia, emmetropia and hyperopia. However, in contrast, Zhang et al. [8], reported a considerably higher WWOP prevalence rate of 15.5%, despite recruiting participants with refractive errors similar to those in our study.

Dhull et al. [21] found a retinal hole prevalence of 0.6%, comparable to our finding of 0.5%, whereas other studies have reported higher prevalence rates ranging from 1.67% to 6.20% [31,32,33]. The prevalence of LD in this study was also relatively low at 0.7%, which is in contrast to previously reported rates varying between 2% and 20% [2]. Since LD is strongly associated with longer ALs and high myopia, this lower prevalence is not unexpected, given that our study had a smaller proportion of participants with high myopia compared to these other studies.

The study reinforces the well-established association between AL, high myopia, and the increased risk of developing PRDs [2,10,32,34]. However, to the best of our knowledge, this is the first study to report the likelihood of a PRD occurrence in a young Australian adult population with diverse ethnic backgrounds and a broad range of ALs and RE. Our logistic regression analysis of RE found that both moderate myopia (OR:3.84, *p* < 0.001) and high myopia (OR:5.37, *p* < 0.001) were found to significantly increase the risk of a PRD being present compared to emmetropes. This is consistent with the findings of Zhang et al. [8], who reported a similar risk for moderate myopia (OR:3.64, *p* < 0.001, but a higher risk for high myopia (OR:10.58, *p* < 0.001). However, unlike Zhang et al. [8], our study did not identify mild myopia as a significant risk factor.

For AL, logistic regression results indicated that eye elongation > 24.50 mm, classified in this study as mild, moderate and high myopia, was also associated with an increased risk of PRD development compared to ALs between 23.50 mm and 24.50 mm (emmetropia). This is interesting, as several studies have typically associated PRDs with ALs > 26 mm [34,35], while Akbani et al. [5], reported the highest occurrence of PRDs in eyes with ALs between ALs of 28 mm and 30 mm.

Clinically, this is an important observation, as while extensive research has established a strong linear correlation between myopia and increased AL (i.e., the higher the myopia, the longer the AL [36]), there can sometimes be discordance between these two parameters. For instance, some individuals with low myopia may have a longer AL than those with moderate myopia. This variation is often due to differences in the refractive components of the cornea and the lens, which can vary from one individual to the next. This highlights the importance of measuring AL alongside RE to better assess the risk of a PRD. If AL measurement is not feasible, reliance on RE becomes necessary. Our results, however, challenge the conventional practice of undertaking a DFE to screen for peripheral retinal changes when RE exceeds −4.00D. Given the significant PRD risk for moderate myopia (OR:3.84, *p* < 0.001), there is a valid argument for reconsidering the threshold for DFE to −3.00D. Bhat [37] also reported a higher prevalence of PRDs, particularly LD, not only in individuals with high myopia but also in those with mild and moderate myopia, further supporting the case for lowering the RE threshold for dilation in young patients.

The optimal age to initiate peripheral retinal screening for the prevention of retinal detachment remains unclear in the current literature. In Australia, most patients undergo a posterior fundus examination as part of a comprehensive eye consultation; however, peripheral retinal screening with dilation for a PRD or RD is generally guided by patient symptoms, such as photopsia, floaters, or by clinical risk factors, including moderate to high myopia. While this study does not directly address the most appropriate age to begin screening, it certainly provides the impetus for further research in this area. This is particularly important given that the risk of a retinal detachment in the general population increases from less than 1% to 9.3% in individuals with refractive error exceeding −5.00D, and rises further to 35.9% when PRDs such as LD are also present [38].

The advancement of UWF imaging has greatly facilitated the ability of eye care practitioners to visualise, evaluate, document and monitor peripheral retinal changes. Midena et al. [39] demonstrated that four-quadrant UWF imaging using the Clarus^TM^ 500 images, performed through a dilated pupil, achieved 99% sensitivity and a 100% specificity in detecting peripheral retinal changes and retinal detachments, compared to binocular indirect ophthalmoscopy (BIO). Similarly, Karatepe Hashas et al. [40], evaluated the use of UWF imaging for detecting retinal breaks with the Zeiss Clarus^TM^ 700, a newer model of the Clarus^TM^ 500 with the same field of view. They also found high sensitivity and specificity in detecting retinal breaks; however, detection rates varied based on the number of images analysed. When only a single image was reviewed, more than 50% of retinal breaks were missed, while at least 75% were detected when two images were analysed. The highest detection rate occurred when six images were reviewed. Since the Zeiss Clarus^TM^ 500 can automatically montage up to six images, expanding the field of view to 267° degrees, future research could investigate whether increasing the number of auto-montaged images from four to six influences prevalence rates and the likelihood of detecting a PRD.

While our study utilised the Clarus™ 500, numerous other investigations have employed the Optos^®^ UWF system for peripheral retinal imaging. Although both devices have their respective advantages and limitations, Matsui et al. [41] reported that the Optos^®^ UWF provided better visualisation of peripheral retina compared to the Clarus^TM^ 500. Conversely, other studies have shown that while the Optos^®^ UWF excelled in imaging the superior temporal region, the Clarus™ 500 outperformed it in capturing the inferior nasal retina [42,43]. It is important to highlight that these comparisons were based on only two montaged images from the Clarus^TM^ 500, whereas our study incorporated four, improving the peripheral retinal coverage.

With respect to PRD prevalence, Sharma et al. [44] reported slightly higher rates of WWOP (6.9%), LD (1.54%), and retinal holes (0.21%) using the Optos^®^ UWF system, relative to our findings. However, their results were derived from a substantially larger dataset of 971 gradable images.

Karatepe Hashas et al. [40] also compared UWF-based detection of retinal breaks with traditional retinal examinations using dilation. Reviewers analysing only UWF images missed retinal breaks in 10–21% of cases, compared to those conducting traditional retinal examinations. Therefore, until UWF imaging can reliably capture the entire peripheral retina, it should be regarded as an adjunct rather than a replacement for BIO in comprehensive ocular assessments. However, in situations where a DFE is not feasible, using UWF imaging to screen at-risk individuals for PRDs could be extremely valuable.

### Limitations

Participants were primarily recruited from a university optometry course. Since higher education attainment has been associated with myopia and the development of longer ALs [28,45], this recruitment approach may limit the generalisability of the findings. Additionally, participants did not undergo a dilated fundus examination using BIO, which, as aforementioned, is widely regarded as the gold standard for evaluating the peripheral retinal fundus, especially when combined with scleral depression. Although the Clarus^TM^ 500 can capture a field of view up to 200° with four montaged images through an undilated pupil, some PRDs may not have been detected, potentially leading to an underestimation of the prevalence.

## 5. Conclusions

PRDs appear to be less prevalent in a mixed population of young Australian adults compared to Asian populations. Validating existing literature, key risk factors for developing PRDs were longer ALs, particularly greater than 24.5 mm, and moderate to high myopic refractive errors. However, most importantly, since AL was found to have a significantly stronger association with PRDs across myopia classifications than RE, incorporating AL measurements alongside RE assessments in clinical practice could enhance eye care practitioners’ ability to predict PRD risk and determine the most appropriate time for retinal evaluation. While a dilated biomicroscopic retinal examination remains the gold standard for assessing the retina, UWF imaging could serve as a suitable alternative when dilation is not possible, enabling early detection of PRDs and reducing the risk of sight-threatening complications, such as retinal detachment.

## Figures and Tables

**Table 1:** Classification criteria for refractive error.

RE Classification *	Values (D)
Hyperopia	>+0.50
Emmetropia	+0.50 to −0.50
Mild myopia	>−0.50 to ≤−3.00
Moderate myopia	>−3.00 to ≤−6.00
High myopia	>−6.00

RE: Refractive error, D = dioptres. * Classification based on [23,24,25,26,27,28].

**Table 2:** Classification criteria for axial length.

AL (mm)	Classification *
<23.50	Hyperopia
≥23.50 to ≤24.50	Emmetropia
>24.50 to ≤25.50	Mild Myopia
>25.50 to ≤26.49	Moderate Myopia
≥26.50	High Myopia

AL: Axial length, mm: millimetres. * Classification based on Khan et al. [29].

**Table 3:** Demographic characteristics.

Parameters	No of Eyes (%)	Mean Age in Years	Mean Age with No PRD in Years	Mean Age with PRD in Years
Gender Male Female	150 (33.9) 292 (66.1)	22.43 ± 11 22.52 ± 3.17 22.38 ± 3.10	22.43 ± 3.15	22.39 ± 2.74

*n* = 442.

**Table 4:** Classification and frequencies of peripheral retinal degenerations.

Peripheral Retinal Degeneration	No. of Eyes (*n*)	Percentage (%)
White Without Pressure	16	3.39
Dark Without Pressure	16	3.39
Lattice Degeneration	3	0.69
Retinal Hole	2	0.45

*n* = 442.

**Table 5:** Frequencies of peripheral retinal degenerations associated with refractive error and axial length.

Parameters	PRD-No. of Eyes (*n*)	Percentage (%)
Emmetropia	1	0.61
Hyperopia	1	2.86
Myopia (all)	34	14.05
Mild Myopia	6	3.95
Moderate Myopia	15	22.06
High Myopia	13	59.09
AL (>24.02 mm)	30	15.23

Emmetropia *n* = 165, Hyperopia *n* = 35, Myopia (all) *n* = 242, Mild myopia *n* = 152, Moderate myopia *n* = 68, High myopia *n* = 22, AL: Axial length (>24.02 mm) *n* = 197, PRD: Peripheral Retinal Degeneration.

**Table 6:** Mean values of refractive error and axial length with and without peripheral retinal degenerations.

Parameters	Eyes with No PRD	Eyes with PRD	*p* ^a^
RE (D)	−1.13 ± 1.94	−4.78 ± 2.33	<0.001
AL (mm)	23.89 ± 1.08	25.46 ± 1.13	<0.001

RE: Refractive error, AL: Axial length, PRD: Peripheral Retinal Degeneration, ^a^ Independent *t*-test, *n* = 442.

**Table 7:** Axial length and refractive error associations with peripheral retinal degenerations.

Parameters	OR	95%CI	*p*
RE			
Emmetropia	Reference		
Hyperopia	1.57	−1.22–4.37	0.27
Mild myopia	1.91	−0.22–4.04	0.079
Moderate myopia	3.84	1.79–5.86	<0.001 ^a^
High myopia	5.47	3.33–7.61	<0.001 ^a^
AL			
Emmetropia	Reference		
Hyperopia	−0.95	−2.60–0.71	0.261
Mild myopia	1.48	0.37–2.59	0.009 ^a^
Moderate myopia	3.00	1.88–4.12	<0.001 ^a^
High myopia	2.82	1.41–4.23	<0.001 ^a^

RE: Refractive error, AL: Axial length, ^a^ Significance *p* < 0.05.

## Data Availability

Data are unavailable due to privacy and ethical restrictions.

## Abbreviations

The following abbreviations are used in this manuscript:

PRD	Peripheral Retinal Degeneration
RE	Refractive Error
AL	Axial Length
UWF	Ultra-widefield
WWOP	White Without Pressure
DWOP	Dark Without Pressure
LD	Lattice Degeneration
CHRPE	Congenital Hypertrophy of the Retinal Pigment Epithelium
OR	Odds Ratio
BIO	Binocular Indirect Ophthalmoscopy
DFE	Dilated Fundus Examination
